# Evaluating Aspirin’s Efficacy for Primary Prevention in Cardiovascular and Cerebrovascular Disease: Insights from a Nationwide Cohort Study

**DOI:** 10.3390/clinpract14040113

**Published:** 2024-07-15

**Authors:** Ki-Hong Kim, Inseok Ko, Jong-Yeup Kim, Dong-Kyu Kim

**Affiliations:** 1 Division of Cardiology, Konyang University Hospital, Daejeon 35365, Republic of Korea; 2 Department of Biomedical Informatics, College of Medicine, Konyang University, Daejeon 35365, Republic of Korea; 3 Division of Big Data and Artificial Intelligence, Department of Otorhinolaryngology—Head and Neck Surgery, Chuncheon Sacred Heart Hospital, Hallym University College of Medicine, Chuncheon 24252, Republic of Korea

**Keywords:** aspirin, myocardial infarction, cerebral infarction, cerebral hemorrhage, gastrointestinal hemorrhage

## Abstract

Background: The effectiveness of aspirin for the primary prevention of cerebro-cardiovascular diseases in Koreans remains unclear. Therefore, we evaluated the preventive effects of low-dose aspirin (equal or less than 100 mg) on cerebro-cardiovascular events. Method: We conducted a retrospective cohort study using the National Sample Cohort dataset. From the 1,106,580 individuals eligible in 2004, we selected 200 individuals (47% male and 22.5% aged 65 or older) who consistently received low-dose aspirin from 2004 to 2013 for inclusion in the aspirin cohort. Participants for the control cohort, who did not use aspirin, were selected through propensity score matching based on variables. Result: We compared the incidences of endpoints (acute myocardial infarction, cerebral infarction, gastrointestinal hemorrhage, and cerebral hemorrhage) between the aspirin group and the non-aspirin group over the 9-year follow-up period. There was no significant difference in the incidence rates of acute myocardial infarction, cerebral infarction, gastrointestinal hemorrhage, or cerebral hemorrhage between the aspirin and non-aspirin groups. Low-dose aspirin for primary prevention in Koreans did not reduce myocardial or cerebral infarctions and did not increase the risk of gastrointestinal or cerebral hemorrhage. Conclusion: Therefore, we suggest that aspirin for primary prevention should be used cautiously and tailored to the individual’s baseline cardiovascular risk.

## 1. Introduction

Aspirin exerts an antithrombotic effect by irreversibly inhibiting platelet activity. This effect results from the inhibition of cyclooxygenase and interference with the biosynthesis of cyclic prostanoids such as thromboxane A_2_ [1]. Nevertheless, aspirin blocks cyclooxygenase, thereby negating the protective effects of PGE2 on the gastric lining, leading to increased risks of ulcers and significant gastrointestinal bleeding within the digestive tract. Hence, it is crucial to meticulously evaluate whether the advantages of administering aspirin outweigh the potential hazards.

Aspirin has been recognized for its preventive benefits in coronary syndrome, leading to the widespread use of low-dose aspirin for the prevention of cerebro-cardiovascular diseases globally [2]. A recent meta-analysis, which included 11 randomized controlled trials, assessed the benefits of low-dose aspirin (100 mg or less per day) for the primary prevention of cardiovascular events in 118,445 participants. This study found that low-dose aspirin reduced the incidence of nonfatal myocardial infarction and nonfatal stroke [3]. However, according to large-scale randomized studies, low-dose aspirin use does not lower the incidence of acute myocardial infarction or stroke compared with a placebo. Moreover, aspirin increased the incidence of major hemorrhage in these studies [4,5]. Another study showed that although aspirin reduced serious vascular events in patients with diabetes, it increased major bleeding events [6]. However, the study predominantly focused on patient groups consisting of White, Black, and Hispanic individuals, leaving the long-term impact of aspirin on Koreans unclear. Therefore, we aimed to assess the impact of low-dose aspirin on preventing cerebro-cardiovascular incidents and bleeding in Koreans without prior cardiovascular disease history, utilizing a representative selection from the National Sample Cohort data of South Korea.

## 2. Materials and Methods

### 2.1. Ethics Statements

This research received approval from the Institutional Review Board of Hallym Medical University Chuncheon Sacred Hospital (2019-05-014). The requirement for written informed consent was waived owing to the use of the National Health Insurance Service—National Sample Cohort dataset, which contains anonymized secondary data intended for research. The research adhered to the ethical guidelines outlined in the Declaration of Helsinki. Furthermore, the authors affirm that the data underpinning the results of this study can be found in the article.

### 2.2. The National Health Insurance Service—National Sample Cohort

Our cohort dataset was a national population-based cohort from the National Health Insurance Service in South Korea. This representative sample database, known as the National Health Insurance Service–National Sample Cohort, was developed due to the impracticality of utilizing the National Health Information Database, primarily because of its extensive size and concerns over privacy breaches [7]. From the 2002 National Health Information Database, which included 46,605,433 individuals, 1,025,340 participants were selected through a random selection process. This sample accounted for 2.2% of the entire eligible population of South Korea in 2002, with the cohort monitored over 11 years until 2013. The database of the National Health Insurance Service—National Sample Cohort encompasses comprehensive medical records, including demographic details (age, gender, income bracket, and mortality) and medical data (prescriptions, treatments for diseases and injuries, rehabilitation services, childbirth, death records, and health promotion activities), all categorized using the *Korean Classification of Diseases*. This classification system closely mirrors the *International Classification of Diseases and Related Health Problems, 10th Revision* (ICD-10).

### 2.3. Aspirin Use

Individuals who consistently received low-dose aspirin (≤100 mg) over 9 years (3287 days) from 2004 to 2013, with prescriptions totaling at least 1620 days, were classified as long-term regular users of low-dose aspirin. Similarly, those who sustained an aspirin intake of ≤100 mg with a prescription duration of at least 180 days within a single year (365 days) were identified as one-year regular aspirin users.

### 2.4. Study Population

Among the 1,106,580 individuals deemed eligible in 2004, a subset of 318,675 adults (aged ≥45 years) was identified for inclusion. To eliminate any prior low-dose aspirin intake, a washout period covering the initial two years (2002–2003) was implemented. During this period, 27,841 participants were documented to have been prescribed low-dose aspirin. The administration of aspirin was specifically for the primary prophylaxis of cerebro-cardiovascular conditions. Patients with a medical history inclusive of acute myocardial infarction, angina pectoris, percutaneous coronary intervention, atrial fibrillation, peripheral vascular disease, cerebral infarction, cerebral hemorrhage, gastrointestinal bleeding, or chronic kidney disease or those undergoing dialysis for end-stage renal disease within the washout timeframe were systematically excluded from the study.

**Figure 1:**
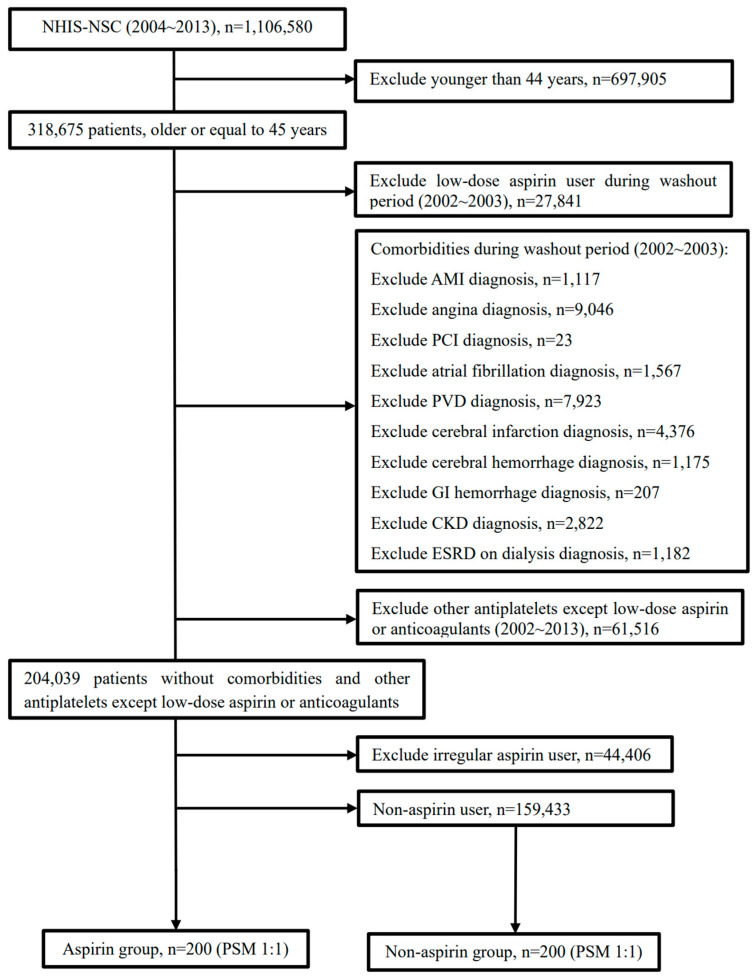
Flow diagram of the inclusion and exclusion criteria for participant selection. We included 200 eligible patients in the aspirin group and 200 patients in the non-aspirin group.

Moreover, individuals who were prescribed antiplatelet agents or anticoagulants except low-dose aspirin during 2002–2013 were omitted from the study cohort. Additionally, those who engaged in inconsistent intake of low-dose aspirin (defined as usage for less than 180 days annually) were excluded. A total of 200 adults who consistently received low-dose aspirin from 2004 to 2013 were selected for inclusion in the aspirin cohort. The selection of participants for the control cohort, who did not use aspirin, was conducted via propensity score matching, considering several independents variables including as age, gender, place of residence, household income, and existing comorbid conditions (Table 1). Ultimately, the study comprised 200 participants in the aspirin cohort and 200 participants in the control cohort. The research methodology and participant selection process are depicted in Figure 1.

### 2.5. Study Endpoints

The primary outcomes of this study were defined as the occurrences of acute myocardial infarction, cerebral infarction, gastrointestinal hemorrhage, and cerebral hemorrhage. To assess these outcomes, the incidence rates in both the aspirin and non-aspirin cohorts were analyzed by calculating person-years at risk, which refers to the period from the initiation of aspirin therapy to the occurrence of any designated endpoint for each participant. To identify traditional cardiovascular risk factors among study participants, diagnoses of hypertension, diabetes mellitus, and dyslipidemia were extracted based on the *Korean Classification of Diseases* codes (hypertension codes I10-13 and I15, diabetes mellitus codes E10-13 and E15, and dyslipidemia code E78). The presence of comorbid conditions was determined by a diagnosis of any of these diseases during the years from 2002 to 2003, establishing a baseline health status before the observation period. This methodology allowed for a comprehensive evaluation of the impact of low-dose aspirin on the prevention of major cardiovascular and cerebrovascular events while accounting for pre-existing health conditions that could affect the outcomes. Through this approach, the study aimed to provide a nuanced understanding of the benefits and risks associated with low-dose aspirin use in a well-defined population, leveraging detailed medical record data to inform the assessment of aspirin’s efficacy in cardiovascular disease prevention.

### 2.6. Statistical Analysis

The incidence rates per 1000 person-years for acute myocardial infarction, cerebral infarction, and major hemorrhagic events such as gastrointestinal hemorrhage and cerebral hemorrhage were calculated for both the aspirin and non-aspirin cohorts. This was performed by dividing the number of patients diagnosed with these conditions by the total person-years at risk. The methodology facilitated a direct comparison of disease occurrence rates between groups. For the assessment of overall disease-free survival rates during the study period, Kaplan–Meier survival analysis was employed. This statistical approach enabled the estimation of the time-to-event data, providing insights into the duration patients remained free of the specified diseases. To investigate the association between aspirin usage and the risk of developing cerebro-cardiovascular diseases or experiencing hemorrhagic events, Cox proportional hazards regression models were utilized. These models were instrumental in computing hazard ratios (HRs) and 95% confidence intervals (CIs), with adjustments made for potential confounding variables. This analysis allowed for the quantification of the relative risk of disease incidence or hemorrhagic events in the aspirin group compared to the non-aspirin group while controlling for other predictor variables. For propensity score matching, 1:1 nearest-neighbor matching was performed without replacement, meaning duplication was not allowed. Several independent variables (Table 1) used in propensity score matching have been adjusted. This adjustment aims to provide more comprehensive information when comparing the two groups, as it allows for the measurement of the effect of aspirin on diseases while considering other control variables together. All statistical analyses were performed using the R statistical software version 3.5.0 (R Foundation for Statistical Computing, Vienna, Austria). The threshold for statistical significance was set at a *p*-value of 0.05, ensuring that findings were evaluated against a standard criterion for determining the likelihood of observed differences being due to chance.

## 3. Results

### 3.1. Differences between the Groups

In the present study, a cohort of 400 participants was analyzed, comprising 200 individuals administered aspirin and 200 participants who did not receive aspirin. The participants were followed up for over 9 years to assess the outcomes. An analysis of baseline demographics and health status indicators, including sex, age, geographical location of residence, income levels, and the prevalence of hypertension, diabetes mellitus, and dyslipidemia, revealed no significant differences between the aspirin and non-aspirin cohorts after propensity score matching. These findings attest to the efficacy of the propensity score matching process in ensuring comparability across the two groups. The comprehensive demographic and clinical characteristics of the participants in each group are presented in Table 1, providing a foundation for the subsequent analysis of aspirin’s impact on health outcomes. This alignment of baseline characteristics is pivotal for the validity of the study’s conclusions, minimizing confounding factors and bolstering the inference that observed differences in outcomes can be attributed to aspirin use.

### 3.2. Cerebro-Cardiovascular Events

**Figure 2:**
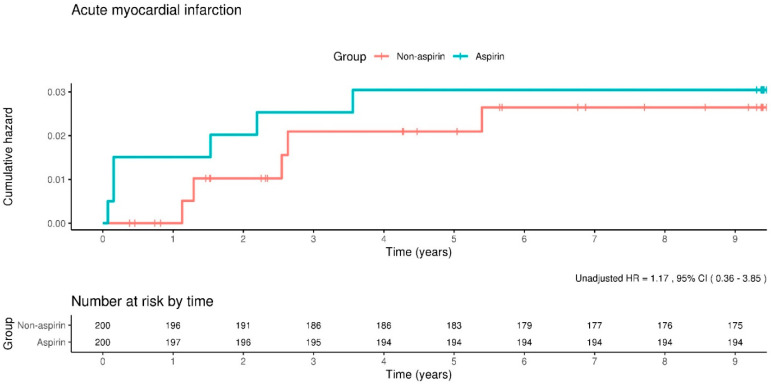
Kaplan–Meier curves showing the incidence of acute myocardial infarction between the aspirin and non-aspirin groups. The rate of acute myocardial infarction does not differ significantly between the groups.

In the present study, the occurrence rate of acute myocardial infarction was 3.2 incidents per 1000 person-years among participants in the aspirin cohort, compared to 2.8 incidents per 1000 person-years in the cohort with aspirin use. Further analysis utilizing adjusted Cox proportional hazard models yielded an HR for acute myocardial infarction of 1.14, with a 95% CI ranging from 0.34 to 3.75, indicating no statistically significant difference in the incidence of acute myocardial infarction between the aspirin and non-aspirin groups. The application of Kaplan–Meier survival analysis techniques provided additional insights, revealing that the risk of experiencing an acute myocardial infarction event was not statistically different between groups, as depicted in Figure 2.

**Figure 3:**
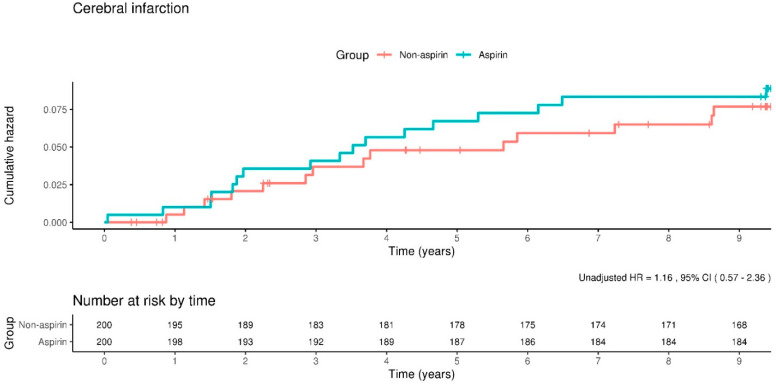
Kaplan–Meier curves showing the incidence of cerebral infarction between the aspirin and non-aspirin groups. The rate of incidence of cerebral infarction does not differ significantly between the groups.

Analysis of the incidence rates for cerebral infarction revealed that in the aspirin cohort, there were 9.2 events per 1000 person-years, compared to 8.0 events per 1000 person-years observed in the non-aspirin cohort. When adjusting for potential confounders, the HR for the incidence of cerebral infarction in the aspirin group was calculated to be 1.13, with a 95% CI of 0.56–2.30. This indicates a lack of significant statistical difference in the occurrence of cerebral infarction between the two study groups. Subsequent examination of the Kaplan–Meier survival curves further corroborated this finding, demonstrating that the incidence rate of cerebral infarction did not exhibit a statistically significant variance between participants in the aspirin and non-aspirin groups, as depicted in Figure 3.

### 3.3. Major Hemorrhagic Events

**Figure 4:**
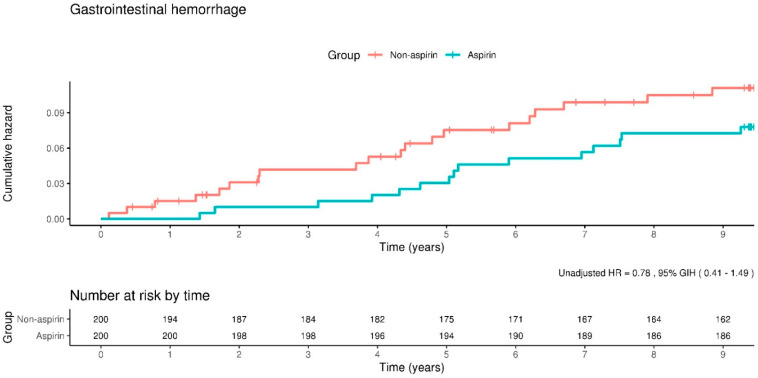
Kaplan–Meier curves showing the incidence of gastrointestinal hemorrhage between the aspirin and non-aspirin groups. The rate of gastrointestinal hemorrhage does not differ significantly between the groups.

The incidence of gastrointestinal bleeding was 9.0 events per 1000 person-years in the aspirin group and 11.6 events per 1000 person-years in the non-aspirin group. Additionally, we found the risk of gastrointestinal bleeding as an adjusted HR of 0.77 (95% CI, 0.40–1.47). Moreover, the Kaplan–Meier curves showed that the incidence rate of gastrointestinal bleeding did not differ significantly between the aspirin and non-aspirin groups (Figure 4).

**Figure 5:**
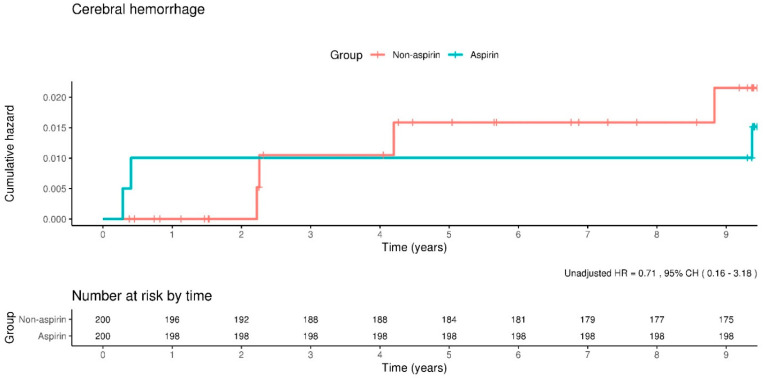
Kaplan–Meier curves showing the incidence of cerebral hemorrhage between the aspirin and non-aspirin groups. The rate of cerebral hemorrhage does not differ significantly between the groups.

Cerebral hemorrhage presents a critical concern in the context of low-dose aspirin therapy, attributed to its strong correlation with elevated mortality risk. In our study, the incidence rate of cerebral hemorrhage was quantified at 1.6 events per 1000 person-years for individuals in the aspirin cohort, in contrast to 2.2 events per 1000 person-years within the non-aspirin cohort. After adjusting for confounding variables, the HR for the occurrence of cerebral hemorrhage among aspirin users was calculated to be 0.70, with 95% CIs of 0.16–3.14. This analysis indicates no significant statistical difference in the incidence of cerebral hemorrhage between the aspirin and non-aspirin groups, as illustrated in Figure 5. These findings underscore the complexity of assessing the risks associated with low-dose aspirin use, particularly in patients with cerebral hemorrhage.

### 3.4. Subgroup Analysis in Patients with Diabetes

We conducted a subgroup analysis on patients with diabetes. No significant differences in endpoints (acute myocardial infarction, cerebral infarction, gastrointestinal hemorrhage, and cerebral hemorrhage) were observed between the aspirin and non-aspirin groups (Table 2).

## 4. Discussion

In 1997, the American Heart Association first enacted a guideline for the primary prevention of cardiovascular disease and stroke and emphasized the importance of lifestyle therapies, such as smoking cessation, cholesterol control, and weight control, to control risk factors [8]. Later, in the 2002 update, low-dose aspirin was recommended for primary preventive purposes only in patients with a higher risk of coronary heart disease (specifically, a 10-year risk of coronary heart disease ≥ 10%). However, the updated guideline did not recommend aspirin for use in patients at risk of bleeding because aspirin increases the risk of gastrointestinal bleeding and hemorrhagic stroke [9]. To date, various cardiovascular disease risk scores have been developed, including the Framingham risk score, which was released in 1998. At present, the American College of Cardiology/American Heart Association pooled-cohort hard cardiovascular disease risk calculator is widely used [10]. In this system, the higher the risk score, the more likely the primary preventive effect of aspirin becomes necessary. Additionally, the ARRIVE study evaluated the effect of aspirin on the primary prevention of cardiovascular events in patients with moderate atherosclerotic cardiovascular disease risk; the mean 10-year atherosclerotic cardiovascular disease risk score at baseline was 17.3%; however, aspirin, compared with the placebo, did not reduce cardiovascular events [4].

In the present study, we found that the prolonged administration of low-dose aspirin did not significantly alter the incidence of acute myocardial or cerebral infarction in Korean individuals without a history of cerebro-cardiovascular conditions. Moreover, the present study findings indicate that aspirin use does not elevate the risk of gastrointestinal bleeding or cerebral hemorrhage within this demographic. While aspirin has been recognized for its efficacy in mitigating the recurrence of cardiovascular events, such as acute myocardial infarction or acute cerebral infarction [2], the advantage of its application in populations without previous episodes of such cerebro-cardiovascular afflictions remains ambiguous. The study’s outcomes contribute to the evolving understanding of aspirin’s preventive utility, emphasizing the need for targeted research to clarify its role in individuals at risk of first-time cerebro-cardiovascular events.

The 2019 American College of Cardiology/American Heart Association guidelines on the primary prevention of cardiovascular disease recommend the consideration of low-dose aspirin (75–100 mg orally daily) for the primary prevention of atherosclerotic cardiovascular disease in selected adults aged 40–70 years who are at higher atherosclerotic cardiovascular disease risk but not at increased bleeding risk [11]. In 2022, the United States Preventive Services Task Force (USPSTF) issued recommendations stating that the initiation of low-dose aspirin for the primary prevention of cardiovascular disease in adults between the ages of 40 and 59 who have a 10-year cardiovascular disease risk of 10% or greater should be considered on an individual basis. Furthermore, the USPSTF advised against the use of low-dose aspirin for primary prevention purposes in individuals aged 60 years and above [12]. This guidance reflects a tailored approach to the use of aspirin, emphasizing the importance of assessing individual risk factors and potential benefits versus risks in the decision-making process for primary prevention of cardiovascular disease. These recommendations underscore the complexity of aspirin’s role in cardiovascular disease prevention and highlight the need for personalized medical advice, particularly in light of evolving evidence regarding its efficacy and safety profile in different age groups and risk categories.

A recently updated meta-analysis showed that aspirin use was associated with a decrease in non-fatal myocardial infarction by 18% and ischemic stroke by 13% but an increase in major bleeding by 50%, intracranial bleeding by 32%, and major gastrointestinal bleeding by 52% [13]. Other meta-analyses have reported similar results [14]. In 2021, the European guidelines reported that aspirin could not be beneficial to patients without established atherosclerotic cardiovascular disease; however, the possibility that the benefit may exceed the risk in high or very high cardiovascular disease risk groups was not excluded [15]. The ASCEND study showed a 12% risk reduction in diabetic patients without evident atherosclerotic cardiovascular disease; however, there was increased major bleeding [6]. Therefore, it was determined that aspirin use may be considered in patients with a very high cardiovascular disease risk without diabetes mellitus. However, further studies are needed in healthy adults aged <70 years at high or very high risk.

The present study showed that aspirin did not decrease acute myocardial infarction. In the ASPREE trial, which enrolled 19,114 healthy persons aged >70 years, aspirin did not decrease the disease [5]. The rate of acute myocardial infarction in the present study (3.2 events per 1000 person–years) was lower than that in the ASPREE trial (4.0 events per 1000 person—years). The present study may have included a lower-risk group than the ASPREE trial did because we included people older than 45 years, whereas the ASPREE study included individuals older than 70 years. The American Heart Association and the American Stroke Association jointly recommend the use of antiplatelet drugs or anticoagulants for secondary stroke prevention in almost all patients [16]. However, in several meta-analyses, aspirin did not show an overall benefit, as it reduced ischemic stroke in the primary prevention of stroke but increased the risk of hemorrhagic stroke [17,18].

The present study showed no difference in the incidence rate of cerebral infarction between the aspirin and non-aspirin groups. The ASPREE trial also showed that aspirin did not decrease the rates of fatal or nonfatal ischemic stroke. In the present study, the incidence rate of cerebral infarction was higher than that in the ASPREE trial, although the present study population was younger than that of the ASPREE trial (9.2 events per 1000 person-years vs. 3.5 events per 1000 person-years). A previous study of stroke statistics in Korea reported that the incidence of ischemic stroke was 229 events per 100,000 person-years [19]. The study population of the present study may have been receiving aspirin continuously because of unidentified risk factors. A recent meta-analysis of bleeding risk associated with aspirin use for primary prevention in adults reported that aspirin increased the risk of major gastrointestinal bleeding by 58% (odds ratio (OR), 1.58; 95% CI: 1.29–1.95) and hemorrhagic stroke by 27% (OR, 1.27; CI: 0.96–1.68) [20]. In addition, the ASPREE trial reported that aspirin increased the risk of major hemorrhage (HR, 1.38; 95% CI: 1.18–1.62; *p* < 0.001) [5]. The ASCEND study showed that aspirin, compared to the placebo, significantly increased the risk of major hemorrhage (4.1% vs. 3.2%; HR, 1.29; 95% CI: 1.09–1.52; *p* = 0.003) [6].

The findings of the present study indicate that a 9-year regimen of low-dose aspirin administration in Korean individuals did not confer an increased risk of gastrointestinal bleeding or cerebral hemorrhage when compared to a cohort with aspirin use. It is noteworthy, however, that individuals who discontinued aspirin due to severe hemorrhagic events might not have been captured within the scope of this study, potentially skewing the observed risk profile associated with aspirin use. Furthermore, it is plausible that participants within the aspirin cohort were concurrently administered gastric mucosal protective agents, such as proton pump inhibitors or histamine receptor antagonists, which could mitigate the risk of gastrointestinal adverse effects and influence the study outcomes. To the authors’ knowledge, there has been limited research specifically exploring the long-term impact of aspirin on gastrointestinal bleeding within the Korean population. This gap underscores the necessity for further empirical investigations to elucidate the relationship between aspirin use and its potential gastrointestinal risks in this demographic. Such studies are crucial for developing comprehensive guidelines that can inform clinical practices regarding the prophylactic use of aspirin, especially considering the balancing act between its cardiovascular benefits and the risk of bleeding complications. The nuanced understanding of these dynamics will contribute significantly to the optimization of aspirin therapy for primary prevention in diverse populations.

As previously mentioned, aspirin damages the gastric mucosa, causing ulcers and increasing the risk of gastrointestinal bleeding. The combination of steroids, non-steroidal anti-inflammatory drugs, and anticoagulants further increases the risk of bleeding. In addition, old age, diabetes, a history of excessive drinking, and decreased renal function are risk factors for gastrointestinal bleeding [21]. H_2_-receptor antagonists and proton pump inhibitors are representative drugs for reducing the risk of bleeding in patients receiving aspirin [22]. Proton pump inhibitors significantly reduce gastric ulcers and gastrointestinal bleeding [23,24]. The H_2_-receptor antagonist was weaker than the proton pump inhibitor but still showed gastrointestinal protective effects. Proton pump inhibitors affect aspirin absorption and metabolism by reducing gastric acidity; thus, they may increase cardiovascular events in patients with acute myocardial infarction [25]. However, a recent large-scale study reported that proton pump inhibitors had no safety problems compared to the placebo in patients receiving aspirin [26]. Therefore, in the present study, aspirin did not increase the risk of gastrointestinal bleeding in the aspirin group compared with the non-aspirin group; however, proton pump inhibitors could be used to prevent gastrointestinal bleeding in the group with a high risk of bleeding.

In the PCI-CURE study, which evaluated the effects of antiplatelet agents in patients undergoing percutaneous coronary intervention, the incidence of major bleeding in patients receiving aspirin was 0.7% [27]. The incidence of gastrointestinal bleeding in patients receiving aspirin after percutaneous coronary intervention in Korea is 3.8% [28]. In the present study, the incidence of gastrointestinal bleeding in the aspirin group was 7.7%, which is twice as high as that reported in other studies. Compared with 6.2 events per 1000 person-years of gastrointestinal bleeding in the ASPREE trial, the bleeding rate of the aspirin group in the present study, with 9.0 events per 1000 person-years, was not higher than that in the ASPREE trial. However, a higher bleeding rate in older patients suggests that the risk of gastrointestinal bleeding may be higher in Koreans than in the Western population, regardless of aspirin use. In the present study, the incidence of a cerebral hemorrhage in the aspirin group was lower than that in the ASPREE study (1.6 events per 1000 person-years vs. 2.5 events per 1000 person-years). This difference may be because the present study included individuals aged >45 years, whereas the ASPREE study included individuals aged >70 years.

Aspirin has been shown to significantly reduce cardiovascular events in diabetic patients without pre-existing cardiovascular diseases [6]. However, in the present study, there were no observed differences in the incidence of acute myocardial infarction, cerebral infarction, gastrointestinal bleeding, or cerebral hemorrhage between two matched groups among diabetes patients. The incidence rates were too low to draw definitive conclusions. Thus, further research is needed to assess the effects of aspirin for the primary prevention of cardiovascular diseases in diabetic patients in Korea. A previous study indicated that taking 10mg of rosuvastatin daily reduced cardiovascular events in individuals at intermediate risk who do not have cardiovascular disease [29]. Additionally, the US Preventive Services Task Force advises clinicians to prescribe statins for the primary prevention of cardiovascular disease in adults aged 40 to 75 who possess one or more risk factors for cardiovascular disease (such as dyslipidemia, diabetes, hypertension, or smoking) and have an estimated 10-year cardiovascular disease risk of 10% or higher [30]. Recently, one study from the EPHESUS database also showed that high-intensity statin with ezetimibe use was lower in Turkish people according to the European Society of Cardiology guidelines and more use of these high-intensity statins with ezetimibe would be helpful to increase primary prevention rate in patients with diabetes [31]. Considering these research findings collectively, aspirin may not demonstrate significant effectiveness for primary prevention of cardiovascular disease. However, statin showed good results in reducing cardio-cerebrovascular events for this purpose, so it would be important to perform statin treatment according to cardiovascular risk in patients without cardiovascular disease.

The present study had some limitations. First, the number of samples included in the study was small and the retrospective nature of the cohort study lacked the feasibility of a prospective study. However, to overcome this problem, we selected two groups in which major socio-demographic independent variables were controlled and analyzed the results. Second, the study did not analyze the additional incidences of hypertension, diabetes, dyslipidemia, or changes in therapeutic medications from the start of the analysis of the participants. As we mentioned in the discussion, this study excluded patients who took P2Y12 inhibitors such as clopidogrel and ticagrelor, which were administered before and after percutaneous coronary intervention; therefore, it is highly likely that many patients with acute myocardial infarction who were treated with percutaneous coronary intervention were excluded from the study. For these reasons, the design of this study prevented us from estimating the baseline cardiovascular risk for considering primary prevention with aspirin. However, to overcome this issue, we included patients who took aspirin regularly until the end of the study period. Thus, the patients who died during the study period were not included in the study. Finally, patient education can impact adherence to therapies and awareness of both therapies and diseases, thereby affecting outcomes. However, the National Sample Cohort dataset lacked information on education levels, preventing us from analyzing this factor; thus, we could not access this information.

## 5. Conclusions

The use of low-dose aspirin for primary prevention of cerebrovascular and cardiovascular diseases in Koreans has not demonstrated a reduction in acute myocardial or cerebral infarction, according to large-scale randomized studies. Moreover, the present study indicates that aspirin does not increase the risk of gastrointestinal bleeding or cerebral hemorrhage. Consequently, the administration of low-dose aspirin for primary prevention in Koreans should be approached with caution. Furthermore, there is a need for more large-scale randomized studies to thoroughly investigate the effects of aspirin on the Korean population.

## Figures and Tables

**Table 1:** Demographics of the study groups (propensity score matched).

Variable	Non-Aspirin (*N* = 200)	Aspirin (*N* = 200)	*p*-Value
Male/Female sex, No. (%)	94 (47.0)/106 (53.0)	94 (47.0)/106 (53.0)	1.000
Age group, No. (%)			1.000
45–64 y	155 (77.5)	155 (77.5)	
≥65 y	45 (22.5)	45 (22.5)	
Residential area, No. (%)			1.000
Capital (Seoul)	58 (29.0)	58 (29.0)	
Other metropolitan cities	43 (21.5)	43 (21.5)	
Other area	99 (49.5)	99 (49.5)	
Household income, No. (%)			1.000
Low (0–30%)	31 (15.5)	31 (15.5)	
Middle (30.1–69.9%)	73 (36.5)	73 (36.5)	
High (70–100%)	96 (48.0)	96 (48.0)	
Hypertension, No. (%)	143 (71.5)	142 (71.0)	1.000
Diabetes mellitus, No. (%)	45 (22.5)	45 (22.5)	1.000
Dyslipidemia, No. (%)	61 (30.5)	61 (30.5)	1.000

**Table 2:** Subgroup analysis in patients with diabetes between aspirin group and non-aspirin group.

	Incidence Rates per 1000 Person-Years					
	Non-Aspirin (*N* = 130)	Aspirin (*N* = 130)	Unadjusted HR (95% CIs)	*p*-Value	Adjusted HR (95% CIs)	*p*-Value
Acute myocardial infarction	4.7	2.3	0.48 (0.09–2.60)	0.392	0.43 (0.08–2.37)	0.333
Cerebral infarction	8.5	15.3	1.79 (0.71–4.49)	0.215	1.63 (0.65–4.12)	0.298
Gastrointestinal hemorrhage	8.4	6.9	0.8 (0.27–2.37)	0.684	0.74 (0.25–2.20)	0.584
Cerebral hemorrhage	1.2	2.3	1.91 (0.17–21.05)	0.598	2.04 (0.19–22.52)	0.560

## Data Availability

The authors confirm that the data supporting the findings of this study are available in the article.
